# Genome-scale cluster analysis of replicated microarrays using shrinkage correlation coefficient

**DOI:** 10.1186/1471-2105-9-288

**Published:** 2008-06-18

**Authors:** Jianchao Yao, Chunqi Chang, Mari L Salmi, Yeung Sam Hung, Ann Loraine, Stanley J Roux

**Affiliations:** 1 Institute for Cellular and Molecular Biology and Department of Mathematics, University of Texas at Austin, Austin, Texas 78712, USA; 2 Department of Electrical and Electronic Engineering, University of Hong Kong, Hong Kong, PR China; 3 Section of Molecular Cell and Developmental Biology, University of Texas at Austin, Austin, Texas 78712, USA; 4 Bioinformatics Research Center, University of North Carolina at Charlotte, Charlotte, NC 28223, USA

## Abstract

**Background:**

Currently, clustering with some form of correlation coefficient as the gene similarity metric has become a popular method for profiling genomic data. The Pearson correlation coefficient and the standard deviation (SD)-weighted correlation coefficient are the two most widely-used correlations as the similarity metrics in clustering microarray data. However, these two correlations are not optimal for analyzing replicated microarray data generated by most laboratories. An effective correlation coefficient is needed to provide statistically sufficient analysis of replicated microarray data.

**Results:**

In this study, we describe a novel correlation coefficient, shrinkage correlation coefficient (SCC), that fully exploits the similarity between the replicated microarray experimental samples. The methodology considers both the number of replicates and the variance within each experimental group in clustering expression data, and provides a robust statistical estimation of the error of replicated microarray data. The value of SCC is revealed by its comparison with two other correlation coefficients that are currently the most widely-used (Pearson correlation coefficient and SD-weighted correlation coefficient) using statistical measures on both synthetic expression data as well as real gene expression data from *Saccharomyces cerevisiae*. Two leading clustering methods, hierarchical and k-means clustering were applied for the comparison. The comparison indicated that using SCC achieves better clustering performance. Applying SCC-based hierarchical clustering to the replicated microarray data obtained from germinating spores of the fern *Ceratopteris richardii*, we discovered two clusters of genes with shared expression patterns during spore germination. Functional analysis suggested that some of the genetic mechanisms that control germination in such diverse plant lineages as mosses and angiosperms are also conserved among ferns.

**Conclusion:**

This study shows that SCC is an alternative to the Pearson correlation coefficient and the SD-weighted correlation coefficient, and is particularly useful for clustering replicated microarray data. This computational approach should be generally useful for proteomic data or other high-throughput analysis methodology.

## Background

Advances in high-throughput technologies, such as DNA microarrays and genome sequencing, have enabled the large-scale exploration of the genome in a way that is systematic, comprehensive, and quantitative. Gene expression profiling has revealed valuable discoveries in basic biological research, pharmacology, and medicine. Currently, clustering has become a popular method for profiling genomic data by which clusters are formed based on the similarity between data points. The points in each specific cluster are similar from each other but different from points outside this cluster.

Clustering methods depend on the measure of pair-wise similarity, the similarity between two points. One commonly used similarity metric is the correlation coefficient between the profiles of the two points, and another commonly used similarity metric is the Euclidean distance. The measure of similarity based on correlation coefficients captures the similarity in shape or pattern of the profiles, and it does not account for the amplitude of the profiles. Scaled versions of any two profiles will have the same correlation coefficient since that of the pair of original profiles, i.e., the amplitude of the profiles, does not affect the correlation coefficient as long as the wave form (shape or pattern) of the profiles is maintained. If the similarity is measured by distance, the amplitude of the profiles does matter. Two profiles with the same pattern but very different amplitudes can have an ideal similarity (essentially the same) when measured by the correlation coefficient, but a very low similarity when measured by the Euclidean distance due to the large difference in amplitudes.

In this study, we focus on clustering based on similarity measured by the correlation coefficient where two genes with similar expression patterns will be considered to be similar regardless of the difference in their amplitudes. Using the Pearson correlation coefficient as the similarity metric, Eisen *et al*. [1] analyzed one of the first genome-wide microarray data sets for the budding yeast *Saccharomyces cerevisiae*. When calculating the gene expression similarity with the Pearson correlation coefficient [1], many studies only averaged the replicates in each experiment [2-4] without taking into account the error in the replicates. Instead of averaging over the replicates, Hughes *et al*. [5] defined an error model which uses a standard deviation (SD)-weighted correlation coefficient (SDCC) to down-weight the gene expression values with high error estimates in their clustering analysis and classified the functions of previously uncharacterized genes by comparing the expression profiles of mutant cells from their *S. cerevisiae *compendium. Using the same correlation, van't Veer *et al*. [6] derived a breast cancer prognosis from the gene expression profile of a primary tumor. In addition, Yeung *et al*. [7] showed that the SD-weighted correlation coefficient improves cluster accuracy and stability to a greater extent than the Pearson correlation coefficient with averaging replicates.

However, the SD-weighted correlation coefficient [5-7] also has disadvantages. The error of measurement is estimated directly by the standard deviation of the replicates, and such an estimate of error can be very inaccurate when the number of replicates is small relative to the number of objects (in this study, genes) [8]. Unfortunately, most of the microarray experiments performed by an academic laboratory employ only small (usually less than 10) number of replicates due to the experimental cost and time concerns, and such a replicate number is much smaller than the amount of genes profiled (usually in the thousands or more). The "Stein phenomenon" [9] suggests that an effective statistical model is needed to deal with replicated microarray data. Here we provide a shrinkage correlation coefficient that considers both the number of replicates and the variance within each experimental group and fully exploits the similarity between the replicated microarray experimental samples. The shrinkage concept is widely accepted as a method to improve the estimation of correlation when the sample size is small [9-11], which is the primary inspiration for this work. We first describe our shrinkage correlation coefficient in generality, and then demonstrate the superiority of our correlation compared to the other two most widely-used correlation coefficients (Pearson correlation coefficient and SD-weighted correlation coefficient) using hierarchical clustering and k-means clustering. Finally we use a recently published analysis of the gene expression changes that occur during the germination of spores of the fern *Ceratopteris richardii *[12] as an example of this shrinkage correlation coefficient.

Other clustering techniques have been used for gene expression analysis. For example, using an analysis of variance (ANOVA) model, Kerr and Churchill [13] applied bootstrapping on a publicly available data set to assess the reliability of clustering results. Ng *et al*. [14] proposed a linear mixed-effects model (LMM) as an extension of the normal mixture model to incorporate covariate information into the clustering process. Tjaden [15] developed a clustering method that is similar to k-means clustering. Using a Bayesian infinite mixture model (IMM), Medvedovic and Sivaganesan [16,17] developed a clustering procedure to incorporate the information on experimental variability into gene expression profiling. IMM measures the similarity using a probabilistic model of the data, and the error between repeated measurements is inherently represented in the model. Instead of forming final clusters directly, a posterior distribution of the possible clusters is generated by Gibbs sampling first. Then the similarity between two data points is measured by the probability of the pair of points being in the same cluster inferred by the posterior distribution of the clustering result. With this measured similarity, final clusters are formed by applying the classical hierarchical clustering. Conceptually, IMM considers the magnitude (rather than pattern) of gene expression profiles and is similar to the Euclidean distance, as noted by Tjaden [15]. In contrast, as a correlation coefficient, our method is based on the pattern of gene expression profiles and is apparently different from IMM when applied to clustering methods.

We stress that our shrinkage correlation coefficient is a correlation instead of a clustering method, and it could be used as a similarity metric in many clustering methods or other circumstances. Therefore, we compare our correlation with two existing widely-used correlation coefficients (Pearson correlation coefficient and SD-weighted correlation coefficient) using hierarchical and k-means clustering. We present our method for better estimating the error in replicated microarrays that cannot be adequately estimated by other correlation coefficients when applying the existing popular clustering methods.

In this paper, we propose a novel correlation coefficient, shrinkage correlation coefficient (SCC). The comparison of SCC with other two most widely-used correlation coefficients using hierachical and k-means clustering shows that SCC is an alternative to the Pearson correlation coefficient and the SD-weighted correlation coefficient and achieves better clustering performance on both synthetic expression data as well as real gene expression data from *Saccharomyces cerevisiae*. We use SCC-based two-dimensional hierarchical clustering to analyze the replicated microarray data of Salmi et al. [12], revealing the novel finding that there are two distinct clusters of genes with shared expression patterns during the early stages of germination of *C. richardii *spores. Findings from this gene expression analysis suggest that some of the mechanisms that control germination in such diverse plant lineages as mosses and angiosperms are also conserved among ferns. We also present the use of singular value decomposition (SVD) [18-20] to uncover the gene-wise bias introduced by experimental artifacts due to comparison of different biological samples and multiple arrays.

## Results

### Shrinkage correlation coefficient (SCC)

Correlation coefficients are computed to measure the similarity between each pair of genes in hierarchical clustering. Assuming that we have a total of *N *arrays consisting of *F *experimental (in this study, time point comparison) groups with *N*(*k*) replicates for the *k*th experimental group, then $N=\sum_{k=1}^{F}N(k)$. Let *G*_*i*,*n*_(*k*) denote the expression level of the *i*th gene for the *n*th replicate in the *k*th experimental group. The mean and variance of the expression of the *i*th gene over the replicates in the *k*th experimental group are defined as ${\bar{G}}_{i}(k)=\sum_{n=1}^{N(k)}G_{i,n}(k)/N(k)$ and $S_{i}^{2}(k)=\frac{1}{N(k)-1}\sum_{n=1}^{N(k)}{\left(G_{i,n}(k)-{\bar{G}}_{i}(k)\right)}^{2}$, respectively.

If the standard deviation (SD) is used as an estimate of the measurement error, then the SD-weighted average expression of gene *i *over the experimental groups is given by

(1) $${\bar{G}}_{i}=\sum_{k=1}^{F}\frac{{\bar{G}}_{i}(k)}{S_{i}^{2}(k)}/\sum_{k=1}^{F}\frac{1}{S_{i}^{2}(k)}$$

and the SD-weighted correlation coefficient is defined as

(2) $$\rho_{ij}^{EW}=\frac{\sum_{k=1}^{F}\frac{\left({\bar{G}}_{i}(k)-{\bar{G}}_{i}\right)}{S_{i}(k)}\frac{\left({\bar{G}}_{j}(k)-{\bar{G}}_{j}\right)}{S_{j}(k)}}{\sqrt{\sum_{k=1}^{F}{\left(\frac{{\bar{G}}_{i}(k)-{\bar{G}}_{i}}{S_{i}(k)}\right)}^{2}\sum_{k=1}^{F}{\left(\frac{{\bar{G}}_{j}(k)-{\bar{G}}_{j}}{S_{j}(k)}\right)}^{2}}},$$

which has been used in the previous studies [5,6]. Since the SD-weighted correlation takes the measurement error into account, it is better than the Pearson correlation coefficient in estimating the correlation between a pair of genes in the case of repeated measurements [5-7]. The concept of applying larger weights on genes with smaller measurement error seems natural and has been demonstrated to be effective. However, the standard deviation may not be the best estimate of measurement error according to the Stein Phenomenon [10,21].

If *F *> 2, the Stein estimation, defined as ${\tilde{S}}_{i}^{2}(k)=(1-\frac{F-2}{D})S_{i}^{2}(k)$ with $D=\sum_{k=1}^{F}{\left(S_{i}^{2}(k)\right)}^{2}$, is better than the standard variance in the sense that it is statistically closer to the real error [21]. The last statement is valid under the assumption that $S_{i}^{2}$(*k*), k = 1,2, ..., *F*, are Gaussian distributed and independent of each other, which can be readily justified using the central limit theorem.

Since the Stein Phenomenon was discovered, several shrinkage estimation methods have been developed to find the optimal estimate of a group of measurement errors. In this work we propose a simple but effective methodology which is mainly inspired by the shrinkage concept [9-11].

Let the real squared measurement errors for the *F *experimental groups be *ψ*_*i*_(1), *ψ*_*i*_(2)...*ψ*_*i*_(*F*). We may estimate these *F *parameters using a high-dimensional model (of dimension *F*). According to statistics theory, the estimates in a high dimensional model will have larger variances compared to those in low-dimensional model (e.g., one dimension) when the same number of data points are available. Furthermore, if the number of data points are very limited (as is typically the case in real examples) the variances of a high-dimensional model may be unacceptably high for practical purposes. To reduce the estimation variance one may map the high-dimensional model for the *F *parameters onto a lower-dimensional restricted submodel. For example we may use the mean of the *F *parameters,

(3) $$\Theta_{i}=\frac{1}{F}\sum_{k=1}^{F}\psi_{i}(k)$$

as a one-dimensional submodel. Then, the estimation variance can be greatly reduced. However, the estimate is biased if we replace *ψ*_*i*_(1), *ψ*_*i*_(2)...*ψ*_*i*_(*F*) by Θ_*i*_.

To summarize, the estimates in the original high-dimensional model have larger variances but are unbiased, and the estimate in the restricted one-dimensional submodel has a smaller variance but is biased. Since neither situation is satisfactory, we will propose a shrinkage error estimate that makes a balance between the above two kinds of estimates.

With the above restricted one-dimensional model (Eq. 3), we have an unbiased estimate of the squared measurement error Θ_*i *_as follows:

(4) $${\bar{S}}_{i}^{2}=\frac{1}{N-F}\sum_{k=1}^{F}\sum_{n=1}^{N(k)}{\left(G_{i,n}(k)-{\bar{G}}_{i}(k)\right)}^{2}=\sum_{k=1}^{F}\frac{N(k)-1}{N-F}S_{i}^{2}(k).$$

Then, we can use a linear regularization model to define a balanced estimate:

(5) $$T_{i}(k)=(1-\lambda_{i})S_{i}^{2}(k)+\lambda_{i}{\bar{S}}_{i}^{2},$$

where *λ*_*i *_∈ [0, 1] is an shrinkage factor that is to be determined according to a chosen optimization criterion. We propose to minimize the risk

(6) $$R(\lambda_{i})=E(\sum_{k=1}^{F}{\left(T_{i}(k)-\psi_{i}(k)\right)}^{2}).$$

Applying the methodology of [11] and [8], the optimal shrinkage factor can be derived as

(7) $${\hat{\lambda }}_{i}=\frac{\sum_{k=1}^{F}\left[\mathrm{var}(S_{i}^{2}(k))-\mathrm{cov}(S_{i}^{2}(k),{\bar{S}}_{i}^{2})\right]}{\sum_{k=1}^{F}E[{\left(S_{i}^{2}(k)-{\bar{S}}_{i}^{2}\right)}^{2}]}.$$

From previous discussion, $S_{i}^{2}$*k*, k = 1,2, ..., *F*, are assumed to be independent of each other, and from the above equation we have

(8) $${\hat{\lambda }}_{i}=\frac{\sum_{k=1}^{F}\left[\mathrm{var}(S_{i}^{2}(k))-\mathrm{cov}(S_{i}^{2}(k),{\bar{S}}_{i}^{2})\right]}{\sum_{k=1}^{F}{\left(S_{i}^{2}(k)-{\bar{S}}_{i}^{2}\right)}^{2}}=\frac{\sum_{k=1}^{F}\left(1-\frac{N(k)-1}{N-F})\mathrm{var}(S_{i}^{2}(k)\right)}{\sum_{k=1}^{F}{\left(S_{i}^{2}(k)-{\bar{S}}_{i}^{2}\right)}^{2}},$$

where var($S_{i}^{2}$(*k*)) is the variance of $S_{i}^{2}$(*k*) estimated by

(9) $$\mathrm{var}(S_{i}^{2}(k))=\frac{N(k)}{{\left(N(k)-1\right)}^{3}}\sum_{n=1}^{N(k)}{\left[{\left(G_{i,n}(k)-{\bar{G}}_{i}(k)\right)}^{2}-S_{i}^{2}(k)\right]}^{2}.$$

To make sure that the shrinkage factor lies between 0 and 1, we define the final shrinkage factor to be

(10) $$\lambda_{i}^{*}=\min(1,\max(0,{\hat{\lambda }}_{i})),$$

and then we obtain the shrinkage estimate of $S_{i}^{2}$(*k*), k = 1,2, ..., *F*, as

(11) $$T_{i}^{*}(k)=(1-\lambda_{i}^{*})S_{i}^{2}(k)+\lambda_{i}^{*}{\bar{S}}_{i}^{2}.$$

Notice that in [8], a related mathematical problem is considered, where it aims to get a shrinkage estimate of a covariance matrix by using a restricted lower dimensional submodel in which the covariance matrix is assumed to be diagonal with common variance. In this work our problem is simpler. We only need to estimate the diagonal elements of the covariance matrix, not the whole matrix. Therefore, our result is different from what is obtained in [8], and is much simpler.

Using $T_{i}^{*}$(*k*), the shrinkage estimate of $S_{i}^{2}$(*k*), the error between the group mean ${\bar{G}}_{i}$(*k*) and the corresponding true expression value can be measured by means of the shrinkage error defined as $\Phi_{i}(k)=\sqrt{\frac{T_{i}^{*}(k)}{N(k)}}$. If we replace *S*_*i*_(*k*) by Φ_*i*_(*k*) in Eqs. 1&2, the shrinkage error-weighted average expression of gene *i *is given by

(12) $${\bar{G}}_{i}^{sw}=\sum_{k=1}^{F}\frac{{\bar{G}}_{i}(k)}{\Phi_{i}^{2}(k)}/\sum_{k=1}^{F}\frac{1}{\Phi_{i}^{2}(k)},$$

and we can define a new shrinkage correlation coefficient for any pair of *i*th and *j*th genes as

(13) $$\rho_{ij}=\frac{\sum_{k=1}^{F}\frac{\left({\bar{G}}_{i}(k)-{\bar{G}}_{i}^{sw}\right)}{\Phi_{i}(k)}\frac{\left({\bar{G}}_{j}(k)-{\bar{G}}_{j}^{sw}\right)}{\Phi_{j}(k)}}{\sqrt{\sum_{k=1}^{F}{\left(\frac{{\bar{G}}_{i}(k)-{\bar{G}}_{i}^{sw}}{\Phi_{i}(k)}\right)}^{2}\sum_{k=1}^{F}{\left(\frac{{\bar{G}}_{j}(k)-{\bar{G}}_{j}^{sw}}{\Phi_{j}(k)}\right)}^{2}}}.$$

If the number of replicates *N*(*k*) is the same for all the experimental (e.g., treatment/condition/time-point) groups, then Φ_*i*_(*k*) = *α*$T_{i}^{*}$(*k*), where *α *= 1/*N*(*k*) is a constant, and hence the shrinkage correlation coefficient (Eq. 13) is effectively weighted by the shrinkage estimate $T_{i}^{*}$(*k*) (i.e., *N*(*k*) has no effect on the shrinkage correlation coefficient). However, if the number of replicates is different for each experimental group, the use of $\Phi_{i}(k)=\sqrt{\frac{T_{i}^{*}(k)}{N(k)}}$ in the shrinkage correlation coefficient provides additional benefits in our method through the weighting *N*(*k*) in a way that agrees with the common practice in statistics [22].

When using a biologically meaningful control sample, e.g., a matched and untreated sample, a zero time point, or the reference sample 24 hr presented in this study, we should use uncentered correlation to keep the impact of the biologically meaningful control sample on the gene expression changes [23]. Therefore, we replace ${\bar{G}}_{i}^{sw}$ and ${\bar{G}}_{j}^{sw}$ with zero in our analysis, and hence Eq. 13 is modified as follows:

(14) $$\rho_{ij}=\frac{\sum_{k=1}^{F}\frac{{\bar{G}}_{i}(k)}{\Phi_{i}(k)}\frac{{\bar{G}}_{j}(k)}{\Phi_{j}(k)}}{\sqrt{\sum_{k=1}^{F}{\left(\frac{{\bar{G}}_{i}(k)}{\Phi_{i}(k)}\right)}^{2}\sum_{k=1}^{F}{\left(\frac{{\bar{G}}_{j}(k)}{\Phi_{j}(k)}\right)}^{2}}}.$$

### Shrinkage correlation coefficient (SCC) is superior in clustering the synthetic and real gene expression data

As shown in Eq. 13, SCC is a type of correlation coefficient which is very useful in gene expression clustering. To assess the effectiveness of a new correlation coefficient in clustering analysis, it is important to compare it with other widely-used correlation coefficents using existing popular clustering methods. In this study, we compared SCC with the two most commonly used correlation coefficients: Pearson correlation coefficient [1] and SD-weighted correlation coefficient [5-7]. We applied these three correlation coefficients on the two most popular clustering methods: hierarchical clustering [24] and k-means clustering [25], and evaluated the performance by comparing the adjusted Rand index [26] generated for each correlation using these two clustering methods. Both synthetic expression data and real yeast expression data [27] were used in this study. The adjusted Rand index is a statistic that has been recently used for the comparison of clustering using different correlation coefficients [14,28-31]. It measures the extent of concurrence between the clustering results and the underlying known cluster structure [32]. The comparison of the adjusted Rand indices generated by different correlation coefficients for the same data set indicates the performance of each correlation. The adjusted Rand index lies between 0 and 1, and a larger index indicates a higher level of agreement between the clustering results and the prior knowledge of functional categories, and further suggests better clustering performance [7,31].

**Figure 1:**
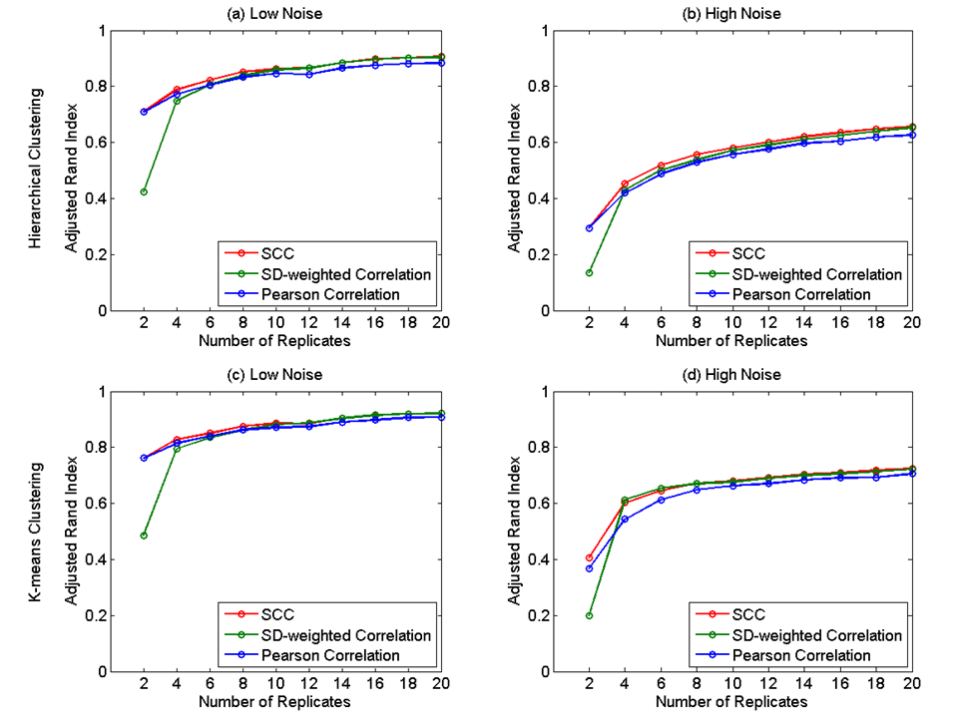
**The performance of the three models indicated by the adjusted Rand index obtained from the synthetic data sets using hierarchical clustering and k-means clustering.** The number of the replicates varies from 2 to 20. Each correlation is represented by a curve: SCC (red), SD-weighted correlation (green), and Pearson correlation (blue). Every data point on a curve is an average adjusted Rand index over 1000 trials of generating and clustering the synthetic data. Hierarchical clustering: (a) Low noise level. (b) High noise level. K-means clustering: (c) Low noise level. (d) High noise level. Error bars are not shown here because, given the scaling of the Figure, they are too small to be graphically depicted after 1000 trials.

Each synthetic data set includes 20 experiments, 2, 4, 6, 8, 10, 12, 14, 16, 18, and 20 replicates for each experiment, and two different levels of noises (low and high). The data sets were generated with predetermined patterns plus low or high level of random noise so that the underlying cluster structure is known. We evaluated the level of agreement between the resulting clusters from each of the three correlations and the known underlying cluster structure by computing the average adjusted Rand index over 1000 randomly generated synthetic data sets. Figure 1a and 1b show the correlation comparisons using hierarchical clustering and Figure 1c and 1d are the results from k-means clustering. As shown in Figure 1a, under low noise level (*α *= 0.5 in Eqs. 15&16), SCC has the same performance as the Pearson correlation coefficient but far better than the SD-weighted correlation coefficient when the replicate number is two. When the number of replicates is four, six and eight, SCC is superior to the other correlations. With the increasing of the number of replicates, the SD-weighted correlation coefficient approaches SCC but both of them still outperform the Pearson correlation coefficient. Figure 1b shows that, when the noise level is high (*α *= 2.5 in Eqs. 15&16), SCC performs the best for all the numbers of replicates. These results suggest that, for synthetic microarray data, SCC is superior to the Pearson correlation coefficient and the SD-weighted correlation coefficient when using hierarchical clustering as it results in the most consistently high adjusted Rand index regardless of noise level and number of replicates.

We also compared the correlations using k-means clustering. Under low noise level (Figure 1c), SCC surpasses the other two correlations when the number of replicates is lower than 12. With the increasing of the number of replicates, the SD-weighted correlation coefficient approaches SCC but both of them still outperform the Pearson correlation coefficient. While the noise level is high (Figure 1d), SCC outperforms the Pearson correlation coefficients for almost all the numbers of replicates. When compared with the SD-weighted correlation coefficient, SCC is better when the number of replicates is smaller than four and close to the SD-weighted correlation coefficient when the number of replicates is four and six. With the increasing of the number of replicates, we noticed that SCC performs almost equally as the SD-weighted correlation coefficient and has a slight advantage when the number of replicates is larger than 14. The k-means clustering results suggests that, SCC is a better choice compared to other correlations when the expression noise level is low, while the noise level is high, SCC is obviously superior to the Pearson correlation coefficient on almost all the numbers of replicates and has slight advantages over the SD-weighted correlation coefficient for most of the numbers of replicates.

**Figure 2:**
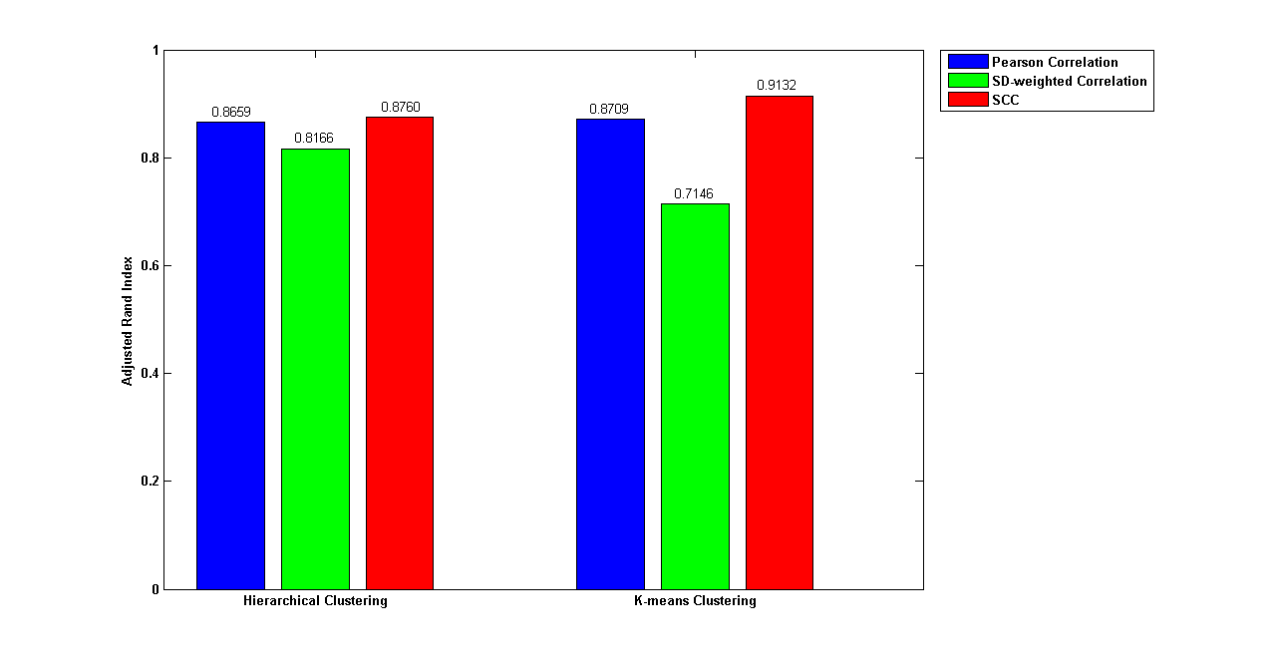
**The performance of the three correlations indicated by the adjusted Rand index obtained from the real yeast expression data using hierarchical clustering and k-means clustering.** Each correlation is represented by a bar: SCC (red), SD-weighted correlation (green), and Pearson correlation (blue). The *y*-axis is the adjusted Rand index.

To further demonstrate the superiority of SCC, we applied the three correlations individually to hierarchical and k-means clustering and computed the adjusted Rand index on the real yeast expression data [27]. This microarray data represent 20 systematic perturbations of the yeast galactose-utilization pathway, and four replicates were performed for each perturbation. Each gene has been annotated in one of four functional clusters in the Gene Ontology [33]. These four clusters are used as the external knowledge. As shown in Figure 2, when hierarchical clustering is used, the adjusted Rand indices for SCC is 0.8760 which is higher than those of the other two correlations (Pearson correlation coefficient: 0.8659; SD-weighted correlation coefficient: 0.8166). When applied to k-means clustering, SCC is also superior to other correlations with the highest adjusted Rand index 0.9132. Since the noise level of this real yeast expression data was not clearly stated and barely quantified in the previous study, we could not determine which is better between the Pearson correlation coefficient and the SD-weighted correlation coefficient. However, this real expression data comparison suggests that SCC is superior regardless of noise level and clustering methods which corresponds to the results with the synthetic data.

### Analysis of the tentative unique gene expression profiles during early development in germinating spores of *C. richardii *by SCC

Spores of *C. richardii *have proved to be an excellent model system to study the basic cellular processes that occur in early gametophyte development, such as gravity sensing and response, sex determination and differentiation, pattern formation, and photomorphogenesis [34]. Using DNA microarrays consisting of 3,840 spotted cDNA clones from an EST analysis, Salmi *et al*. [12] monitored the mRNA levels for 3,207 tentative unique genes (TUGs) of *C. richardii *over the first 48 hr of gametophyte development. TUG expression in the spores was evaluated at 0, 6, 12, 24 and 48 hr after spores were exposed to continuous white light. This developmental period includes initiation of germination at 0 hr, the production of a detectable polar calcium current that peaks at 6–12 hr, fixation of the polarity of more than half of cells by gravity at 12 hr, migration of the nucleus at 24 hr, and a polar cell division at 48 hr [35].

The data analysis of Salmi *et al*. [12] focused on identification of differentially-expressed genes between time points and did not attempt to identify upward or downward trends in the time course data. This analysis did not discover and filter out the underlying biases associated with experimental artifacts due to comparison of different biological replicates and prints of arrays to facilitate further gene expression profiling. Moreover, this analysis did not organize expression patterns into biologically meaningful profiles through the whole time course experiments by assimilating the patterns of gene expression. A more thorough analysis of these microarray expression data that focuses on characterizing the entire set of transcripts temporally and displaying them graphically would promote a better understanding of cellular processes underlying early gametophyte development in ferns.

In total, we analyzed 34 arrays with biological replicates of four different developmental time point comparisons: nine replicates of 0:24 hr, eight of 6:24 hr, nine of 12:24 hr, and eight of 48:24 hr. The 34 arrays were used to generate a total of 34 data columns in which each column was treated independently rather than averaging the replicates.

The initial selection retained non-flagged spots for which the within-spot pixel-to-pixel correlation of intensities is > 0.5 and the sum of median (635/532) signal intensities is >150. These non-flagged spots were also well-measured in at least 80% of the array. In this selection, 39% of the TUGs were filtered out prior to further analysis. Selecting for TUGs with a known accession number in GenBank removed a further 4% of the TUGs. Selection for a fluorescence ratio of at least 1.5-fold greater than the geometric mean ratio for the TUGs examined in at least two arrays of any time point comparison removed 41%. The resulting data set (see Additional file 1) for the experiments analyzed included tabulation of the log_2_ratios of gene-expression levels for 572 TUGs. In the entire 34 arrays there were 137 TUGs for which there were no missing data. A total of 1,152 expression ratios were missed, accounting for only about 6% of the total data set we analyzed. After the imputation of the missing data with the K-nearest neighbors (KNN) method [36], the data set was normalized array-wise with the mean of 572 TUGs expression ratios of each array set as zero and the standard deviation as one.

We first uncovered ten samples that have poor correlation to other samples of their time point group by applying unsupervised agglomerative hierarchical array clustering and SVD (see Additional file 2). These ten arrays, which likely represent experimental artifacts, were removed from the data set, and the new data set (see Additional file 3) was used to tabulate the log_2 _ratios of gene-expression levels for 572 TUGs. Of these TUGs, 151 have no missing data in the entire 24 arrays. A total of 1020 data were missed, accounting for only 7.4% of the adjusted data set. The imputation of the missing data was performed by the K-nearest neighbors (KNN) method [36] with the average value of the nearest 10 neighbors (K = 10). We further normalized this new data set by adjusting the mean of 572 TUGs expression ratios of each array to zero and the standard deviation to one.

**Figure 3:**
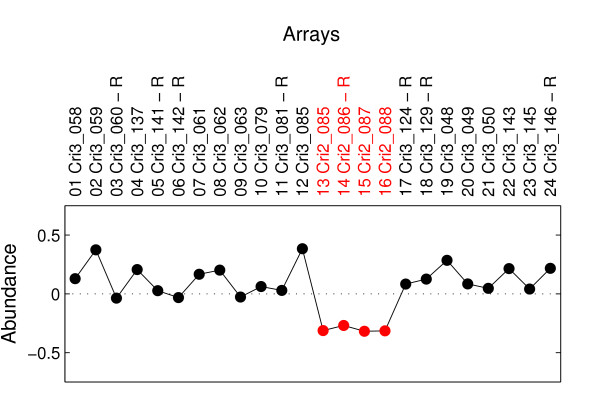
**Gene-wise bias (Eigengene 5) associated with the two prints of arrays.** The abundance of Eigengene 5 in each of the 24 arrays with the arrays in the order obtained in Additional file 3. The 24 dots denote all of the arrays: Cri2 arrays (*red*), Cri3 arrays (*black*). Array names are similarly color coded.

In the new data set, the expression profile of the 12:24 hr time point comparison group was measured by two prints of arrays: four replicates hybridized on the Cri2 arrays, and two on the Cri3 arrays. Cri2 and Cri3 arrays were the same except printed on different days. In attempt to identify and correct the gene-wise bias introduced by the two prints of arrays, we carried out SVD. This technique has previously been used to detect and correct the artifact in the data set that was caused by different types of arrays (22 K vs. 42 K) [37] or sampling from cultures with slightly different periods [38]. We reduced the new "*TUGs *× *Arrays*" space to the "*Eigenarrays" *space that spans the space of the array expression profiles and the "*Eigengenes" *space that spans the space of the gene expression profiles. Eigengene 5 was discovered to be exactly correlated with the gene-wise bias (Figure 3). We sorted the abundance of this eigengene in each of the 24 arrays, and found a perfect correlation between the abundance and the print of array (Figure 3). All of the four Cri2 arrays show negative abundances, whereas the Cri3 arrays all have non-negative abundances.

**Figure 4:**
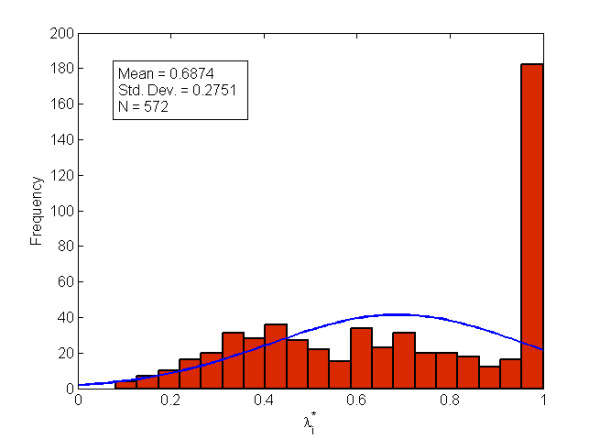
**Histogram of optimal shrinkage factor**$\lambda_{i}^{*}$. The mean, standard deviation, and the total number of $\lambda_{i}^{*}$ are shown in the left upper corner of the histogram.

**Figure 5:**
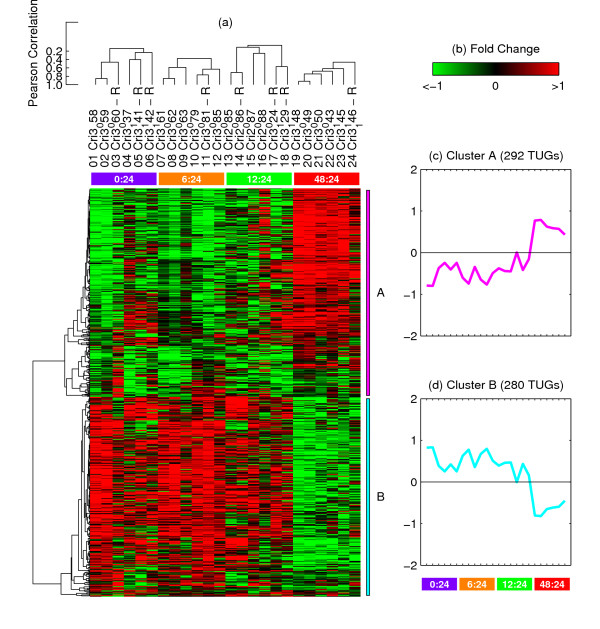
**TUG expression profile in the early stages of gametophyte development of *C. richardii *by SCC.** (a) Unsupervised two-dimensional hierarchical clustering. Data are presented in a matrix format: each row represents an individual TUG, and each column corresponding to an experimental sample. Each expression measurement represents the normalized log_2 _ratio of fluorescence from the hybridized experimental sample to a reference sample. Normalized TUG expression ratios are depicted by a pseudocolor scale with red indicating positive expression above the reference, black indicating equal expression as the reference, and green indicating negative expression below the reference. The horizontal colored boxes delimit four pairwise time point comparison groups: 0:24 hr (*violet box*), 6:24 hr (*orange box*), 12:24 hr (*green box*), and 48:24 hr (*red box*). The scale to the left of the dendrograms depicts the Pearson correlation coefficient represented by the length of the dendrograms branches connecting pairs of nodes. (b) The fold change scale extends from fluorescence ratios of -1 to 1 in log_2_units. (c) Average expression profiles of Cluster A, computed by averaging the log_2_(Cy5/Cy3) ratios. (d) Average expression profiles of Cluster B, computed by averaging the log_2_(Cy5/Cy3) ratios.

We filtered out the gene-wise bias from the data set (see Additional file 3) by substituting Eigenexpression 5 (the diagonal entry of *S *in Eq. 1 of Additional file 2) with zero, and reconstructed the final data set with Eq. 1 of Additional file 2. Subsequently, this final data set (see Additional file 4) was analyzed by the unsupervised two-dimensional agglomerative hierarchical clustering. We first calculated the optimal shrinkage factor $\lambda_{i}^{*}$ with Eq. 10. Since the number of replicates is six (a moderate number) for each time point comparison after we filtered out ten low-quality arrays, $\lambda_{i}^{*}$ is significantly different from 0 and 1, and has an average value of 0.69 (Figure 4). This indicates SCC is effective in our analysis, and neither the Pearson correlation coefficient nor the SD-weighted correlation coefficient should be used here. Therefore, we applied SCC to the gene clustering, and the Pearson correlation coefficient [1] to the array clustering. The TUG expression profiles during the early stages of gametophyte development of *C. richardii *are shown in Figure 5. The 572 TUGs are clearly clustered into two distinct groups: Cluster A with 292 TUGs, and Cluster B with 280 TUGs.

We performed functional analysis of clusters A and B using Gene Ontology annotations transferred from putative *A. thaliana *homologs of individual TUGs, as identified by blastx analysis described previously [12]. Using a standard over-representation analysis as implemented in the ErmineJ software [39], we examined the clusters to identify Gene Ontology terms that appeared unusually often among the TUGs in each cluster, relative to the full complement of TUGs represented on the array.

We found that Cluster A, which includes genes that are up-regulated during the first 48 hours following germination, were significantly enriched with genes annotated with the GO term RNA-binding. The cluster contained TUGs that had high homology to *A. thaliana *proteins At4g32720.1 (CriU1545, CriU226) and At2g05120.1 (CriU2095). At4g32720.1 contains an RNA recognition motif, and At2g05120.1 contains a region matching a nucleoporin Pfam motif. Both are annotated with the term "RNA export from nucleus," suggesting that their *C. richardii *counterparts may also be involved in RNA processing and export.

Over-representation analysis of Cluster B, which includes genes that are down-regulated during the same period of development, revealed fourteen enriched terms. These included terms related to signal transduction (protein phosphatase type 2C activity, abscisic-acid mediated signaling, hormone-mediated signaling), transport, biosynthetic activities (water-soluble vitamin biosynthesis), oxidative phosphorylation, and response to oxidative stress. A full list of terms is given in Table 1.

**Table 1:** GO categories significantly (FDR < 0.10) enriched among genes belonging to SCC Clusters A and B.

**GO id**	**Term**	**Genes**	**p value (ORA)**
**Cluster A**			
GO:0050658	RNA transport	5	0.0002

**Cluster B**			
GO:0009615	response to virus	8	0.0001
GO:0008047	enzyme activator activity	5	0.0002
GO:0030312	external encapsulating structure	5	0.0002
GO:0017077	oxidative phosphorylation uncoupler activity	5	0.0002
GO:0008287	protein serine/threonine phosphatase complex	9	0.0003
GO:0015071	protein phosphatase type 2C activity	6	0.005
GO:0016311	dephosphorylation	11	0.001
GO:0042221	response to chemical stimulus	48	0.0011
GO:0005730	nucleolus	8	0.0022
GO:0042364	water-soluble vitamin biosynthesis	8	0.0022
GO:0009738	abscisic acid mediated signaling	8	0.0022
GO:0006810	transport	92	0.0022
GO:0005886	plasma membrane	25	0.0027
GO:0009755	hormone-mediated signaling	14	0.0035

GO id: the Gene Ontology identifier for each over-represented Gene Ontology terms. Term: the name of the term. Genes: Number of genes on the array with the GO annotation term in column 1. P value: The unadjusted p value from the over-representation analysis (ORA) test.

In addition, we noticed that the final data set (see Additional file 4) analyzed here was generated by filtering out ten low-quality microarray samples and one gene-wise bias from the original 34 arrays. Therefore, it will be reasonable to obtain similar clusters based on this final data set by applying the three correlations (SCC, Pearson correlation coefficient and SD-weighted correlation coefficient) to hierarchical or k-means clustering (data are not presented here). Since SCC is superior in clustering synthetic and real expression data as demonstrated above, we presented here the application of SCC to analyze our own microarray data.

## Discussion

In this work we have shown that a new correlation coefficient, shrinkage correlation coefficient (SCC), can be used as a similarity metric in clustering replicated microarray data generated by most academic laboratories and derive new information pertaining to gene expression patterns. Using hierarchical and k-means clustering, we compared SCC with the two most widely-used correlation coefficients: Pearson correlation coefficient [1] and SD-weighted correlation coefficient [5-7]. The adjusted Rand index comparison showed that SCC enables improved clustering performance for both synthetic and real expression data. Using the SCC-based hierarchical clustering algorithm, we discovered two distinct clusters of genes during the germination of *C. richardii *spores. Functional analysis suggests that some of the mechanisms that control germination in such diverse plant lineages as mosses and angiosperms are also conserved among ferns.

### SCC is a robust correlation coefficient

Correlation coefficient is crucial in cluster analysis to determine the similarity between two objects (in this study, genes) and further classify the objects into different groups. When the Pearson correlation coefficient was first applied for clustering gene expression [1], the replicates of each treatment group were simply averaged without considering the underlying error. As the importance of the error information was discovered [5], more and more studies use standard deviation as the error estimate when clustering gene expression with the help of correlation coefficient. Using the SD-weighted correlation coefficient, they down-weighted the gene expression values with high error estimates in microarray analysis [5-7]. However, the SD-weighted correlation coefficient is still not statistically efficient for analyzing replicated microarray data and the use of standard deviation as the error estimate exhibits serious defects when the number of replicates is small [8].

Commonly, the number of microarray replicates that are performed by most academic laboratories is usually less than 10 due to the experimental cost and time concern. The "Stein phenomenon" [9] states that when the number of data samples (in this study, biological replicates) in each experimental group is relatively small, a better estimate of the error of any individual experimental group could be obtained by shrinkage that considers all experimental groups. To avoid inaccuracy introduced by the small number of replicates, a better estimate of the error in the replicates can be obtained by shrinkage estimate. Our shrinkage correlation coefficient (Eq. 13) can be regarded as a generalized definition of correlation coefficient for the expression of a pair of genes with replicates. It takes into consideration both the number of replicates and the variance within each treatment comparison.

SCC can be reduced to other definitions of correlation coefficients when the shrinkage factor $\lambda_{i}^{*}$ is set to some special values. For example, under the condition that the numbers of replicates are equal for all the *F *experimental groups, SCC is equivalent to the Pearson correlation coefficient when $\lambda_{i}^{*}$ = 1, and to the SD-weighted correlation coefficient when $\lambda_{i}^{*}$ = 0. Therefore, the SD-weighted correlation coefficient and the Pearson correlation coefficient are just two extreme cases of SCC. The correlation coefficient with optimal shrinkage is an alternative to these two extremes and is superior to them when 0 <$\lambda_{i}^{*}$ < 1. By using the shrinkage factor $\lambda_{i}^{*}$, we obtain an optimal estimate of the error in the replicates and, accordingly, better estimates of the similarity between any pair of genes.

We would argue that in SCC, the use of the shrinkage error $\Phi_{i}(k)=\sqrt{\frac{T_{i}^{*}(k)}{N(k)}}$ as a weighting provides a better correlation measure for two reasons. First, $T_{i}^{*}$(*k*) is superior to the standard deviation as an estimate of the measurement error, and secondly, the inclusion of *N*(*k*) takes the size of an experimental group into account as an assessment of the reliability of the group mean. The benefit of *N*(*k*) disappears if all experimental groups are of the same size. For example, in our *C. richardii *microarray data analysis, the number of replicates happens to be equal for each time point comparison after filtering out ten low-quality arrays. In such an analysis, the use of $\Phi_{i}(k)=\sqrt{\frac{T_{i}^{*}(k)}{N(k)}}$ and the use of $\Phi_{i}(k)=\sqrt{T_{i}^{*}(k)}$ are equivalent, since either choice leads to the same value of shrinkage correlation in Eq. 13. Figure 1a, b and 1c shows that SCC is superior to the other models regardless of the number of replicates. In Figure 1d, SCC also has advantages for most of the numbers of replicates. Therefore, SCC offers utility to most replicated microarray data sets.

Using the Stein shrinkage concept [9,10], a shrinkage estimator for gene-specific variance components was proposed to construct a *F*-like statistic that has been used in a linear mixed ANOVA (analysis of variance) model [40], and a shrinkage estimator of the mean used in the clustering similarity metric was developed for genome-wide expression data analysis [41]. This shrinkage ANOVA model and the shrinkage mean could be combined with our SCC for analyzing expression data in future studies.

### Results of functional analysis

The stringent quality control filtering followed by novel cluster analysis methodology of microarray data on *C. richardii *early development produced two distinct clusters of TUGs. As shown in Figure 5, the TUGs represented in SCC Cluster A increased expression during the first 48 hours following germination, while TUGs in Cluster B decreased expression during the same period. An analysis of GO terms associated with TUGs in Cluster A reveals that only one term (RNA transport) is over-represented among GO annotations associated with Cluster A TUGs relative to the other TUGs assayed in the microarrays. This suggests that the TUGs in Cluster A represent a cross-section of many different types of genes, perhaps reflecting a general "ramp-up" in multiple biological functions, along with an increase in RNA processing as the spore activates embryonic transcription.

Previous results from other systems suggests that genes associated with the term RNA transport may be related to the process of establishing and maintaining cellular polarity in the early stages of germination of *C. richardii *spores. In the filamentous fungus *Aspergillus nidulans*, mutation of swoK, a gene that encodes a protein with an N-terminal RNA recognition motif that causes cells to swell and lack the normal polarity maintained fungal hyphal cells [42]. The swoK protein appears to function in both mRNA maturation and nuclear export of mRNAs.

By contrast, over-representation analysis of the Cluster B TUGs, which are down-regulated during the 48 hr time course, reveals an enrichment of several GO terms, suggesting that the Cluster B TUGs represent a more specialized set of genes involved in functions and processes required during early development of *C. richardii*. Cluster B consists of genes that are down-regulated during the earliest stages of spore germination, starting with the initiation of germination by light (0 hr) through the first two days of development, when the first cell division occurs. These are likely to include transcripts that were present in the dormant spore but decline in abundance in the germinating spore. This population is likely to encode proteins involved in maintaining the dormant condition. Once germination begins, genes responsible for maintaining the dormant condition of the spore would need to be down-regulated to allow for the transition from dormant metabolism to active growth and development.

Careful examination of the Cluster B genes and their associated GO terms reveals some interesting patterns. One of the most notable findings from this study is that genes involved in abscisic acid mediated signaling are overrepresented among genes down-regulated in the first 48 hr of spore germination. Abscisic acid (ABA) is a plant hormone known to be involved in the process of establishing and maintaining dormancy in angiosperm seeds [43,44] and moss spores [45]. ABA has been previously shown to be involved in another aspect of *C. richardii *development. Sex determination of *C. richardii *gametophytes is regulated in part by ABA [46].

The process of seed germination in *Arabidopsis *involves a decrease in the endogenous levels of abscisic acid [43], and inhibiting ABA biosynthesis by treatment with fluridone caused *Nicotiana plumbaginifolia *seeds that should be physiologically dormant (D) to germinate at the same rate as seeds that are in a physiologically non-dormant (ND) condition [44]. The more general term hormone-mediated signaling (a sub-set of which would be abscisic acid-mediated signaling) is also over-represented among the TUGs in Cluster B. This term annotates TUGs predicted to be involved in ABA-related pathways as well as other hormone-related processes, notably signaling pathways mediated by gibberellic acid. Hormone-mediated regulation of the process of germination has been well studied in angiosperm seeds, including ABA involvement in the maintenance of dormancy, and the germination activating role of gibberellin. The process of germination in angiosperm seeds involves extensive hormone-mediated signaling [47] so it is not surprising to find this category of genes implicated in fern spore germination, as well.

The involvement of ABA in germination is not unique to angiosperm seeds. In moss cells that have differentiated into spores, removal of ABA causes these cells to germinate and develop into new filamentous cells [48]. In the classical view of hormone signaling in eukaryotic organisms hormones are thought of as the chemical substances produced in one part of the organism that serves as a signal to another part of the organism. In this paradigm it would seem unusual for a plant hormone to function within the single cell of the *C. richardii *spore. However, an ABA receptor involved in seed germination has been characterized that is not plasma membrane localized [49], the Arabidopsis gene CHLH (genomic locus At5g13630). This receptor functions inside of cells and it could, in principle, respond to intracellular changes in the level of ABA to regulate physiological changes within single cells. The CHLH "receptor" gene encodes a subunit of the Mg-chelatase complex that is an integral part of chlorophyll biosynthesis, and is involved in plastid-to-nucleus signaling. The process of producing a highly resistant, dormant stage of plant reproductive cycles (i.e. a spore or seed) is conserved among all major plant lineages. Given the intercellular localization and functioning of an ABA receptor that mediates germination in *Arabidopsis *seeds, and the documented role of ABA in the germination of various spores, it is plausible that this signaling pathway is similar in fern and moss germination.

One of the well-documented mechanisms that regulates ABA signaling in seed germination is dephosphorylation of regulatory proteins by protein phosphatases, particularly type 2C protein phosphatases (PP2C) [50,51]. In *Fagus sylvatica *the expression of a PP2C is directly regulated by ABA in dormant seeds [52]. Additionally, dephosphorylation of actin by protein phosphatases has been implicated in the germination of the plasmodium *Physarum sclerotium *[53] and *Dictyostelium discoideum *[54]. The biological process of dephosphorylation was included as an activity that is over-represented in the cluster of genes down regulated during germination, and this category includes genes likely to encode PP2C enzymes. The prediction from our clustering results that ABA-regulated signaling enzymes like protein phosphatases are involved in fern spore germination is plausible in light of the conservation of this pathway in other plant species.

Annotation of genes in the down-regulated cluster identified three cellular localization categories, including external encapsulating structure, nucleolus, and plasma membrane. Of these three categories, the down regulation of nucleolar associated genes is similar to a process observed in angiosperm seed germination. In *Zea mays *seeds, nucleolus-associated bodies are present in the cells of dry seeds, but after 24 h of imbibition these nuclear bodies have decreased significantly [55]. Although *C. richardii *spores are a useful model system for the study of gravity perception in a single cell, we did not identify any genes in this analysis of early development that are obviously involved in the process of gravity perception or in early signaling steps of the gravity response.

## Conclusion

In summary, we have developed a robust correlation coefficient, shrinkage correlation coefficient (SCC), which is an alternative to the Pearson correlation coefficient and the SD-weighted correlation coefficient, and particularly useful for clustering replicated microarray data generated by most academic laboratories. We have shown the superiority of SCC by the adjusted Rand index comparison on both synthetic and real expression data using hierarchical and k-means clustering. We apply SCC to successfully identify distinct clusters of genes during *C. richardii *early development. We also present the use of SVD to uncover the gene-wise biases introduced by experimental artifacts due to comparison of different biological replicates and prints of arrays. This computational approach is not only applicable to DNA microarray analysis but is also applicable to proteomics data or any other high-throughput analysis methodology.

## Methods

### Hierarchical clustering and k-means clustering

The agglomerative hierarchical clustering algorithm [24] was used in this study. It starts with individual objects. The most similar objects are clustered together, and then these initial clusters are merged according to their similarities. This hierarchical clustering algorithm is based on the average linkage method [56]. In our two-dimensional cluster analysis, gene clustering and array clustering are performed independently without interfering between the two dimensions. K-means [25] is a classic clustering algorithm which assigns each point to the cluster whose centroid is nearest. In our study, the clustering results of hierarchical clustering were used to compute the initial centroids to start k-means. All of the clustering analyses were implemented with MATLAB (MathWorks, Inc., Natick, MA).

### Synthetic expression data

Following the convention of [7], we generated synthetic microarray data which include six clusters of genes and 20 experiments. Each cluster consists of 66 or 67 genes. We use the subscripts *i*, *j*, *k *and *m *to denote the gene number, the experiment number, the replicate number and the cluster number, respectively. The first four clusters of genes follow periodic functions plus some noises:

(15) $$g_{ijk}=\sin\left(k\omega_{m}/n+\phi_{m}\right)+\alpha \sigma_{i}{\hat{\sigma }}_{j}x_{ijk}.$$

The fifth and sixth clusters of genes follow linear functions plus some noises:

(16) $$g_{ijk}=\pm k/n+\alpha \sigma_{i}{\hat{\sigma }}_{j}x_{ijk}.$$

In the above equations, *g*_*ijk *_is the gene expression for the *i*th gene, *j*th experiment and *k*th replicate. The parameters *ω*_*m *_and *φ*_*m *_represent the wavelength and the shift for the *m*th cluster (*m *= 1,2,3,4). They are random variables drawn from uniform distributions on [0.5*π*, 5*π*] and [0,2*π*], respectively. *n *= 20 is the total number of experiments. The parameters *σ*_*i *_and ${\hat{\sigma }}_{j}$ represent the error levels for the *i*th gene and *j*th experiment. They are random variables drawn from uniform distributions on [0.2, 1.2]. The parameter α is chosen *a priori *and determines the overall noise level of this set of synthetic data. The random variable *x*_*ijk *_is drawn from a standard normal distribution.

### Real expression data from *Ceratopteris richardii*

Data analyzed here were collected from spotted cDNA microarrays produced by our lab. TUG (tentative unique gene) expression changes in *Ceratopteris richardii *were studied during the emergence from dormancy over the first 48 hr of spore germination using microarrays representing an estimated 3,207 distinct genes from this organism [12]. Four different pairwise developmental time point comparisons were conducted with a minimum of eight replicates for each comparison: 0 vs. 24 hr, 6 vs. 24 hr, 12 vs. 24 hr, 48 vs. 24 hr. The reference sample was 24 hr for these experiments. Total RNA samples from each time point were labeled during reverse transcription with one of the fluorescent Cy5 (red) or Cy3 (green) dyes (Amersham Biosciences, Buckinghamshire, UK).

Experimental design, including probe synthesis, hybridization conditions and array scanning can be found in a published protocol [12]. Dye-swap experiments (biological repliates) were included for all time point comparisons. Raw data, array images, settings, grid files, red/green scan files, compiled tabular data, detailed protocols are publicly available from the Longhorn Array Database (LAD) [57].

It should be noted that for the 12:24 hr time point comparison group, two prints of arrays (Cri2 and Cri3) were used for hybridization. These arrays were the same except printed on different days. After four replicates were conducted on the Cri2 arrays, the new Cri3 arrays were used for the remaining five replicates as described [12].

### *Ceratopteris richardii *data retrieval and missing value imputation

Spots with aberrant measurements due to array artifacts or poor quality were manually flagged, and spots contaminated with dust or fluorescent specks were excluded from further analysis. The log_2 _of background-subtracted, normalized median spot intensities of ratios from the two channels (Cy5/Cy3) were retrieved from LAD [57] after filtering out spots that had weak signal intensities based on the following criteria: the regression correlation value between the signal intensities in the two channels (Cy5 and Cy3) across all pixels was required to be greater than 0.5, and the sum of median intensities for the two channels was required to be greater than 150. Spots that meet the above criteria had to make up at least 80% of the array for it to be included in further analysis. To focus this analysis on the TUGs with the greatest changes in expression, we selected TUGs whose fluorescence intensity ratio (in at least two replicate arrays of any time point comparison) differed by ≥ 1.5-fold from their geometric mean ratio across the entire set of arrays.

Any missing values in arrays included in analysis were imputed by the K-nearest neighbors (KNN) algorithm [36] with the average value of the nearest ten neighbors (K = 10). The Euclidean distance was used to determine the nearest neighbors for a given gene. These imputed values were used throughout the analysis but were left blank in the primary data tables.

### Functional analysis using Gene Ontology

Over-representation analysis of Gene Ontology annotations associated with clusters was performed using the "ORA" analysis option in version 2.12 of the ErmineJ software [39]. Briefly, this analysis uses a re-sampling approach to compute empirical p values for each GO annotation associated with cluster members, followed by multiple hypothesis testing correction using the Benjamini-Hochberg false discovery rate (FDR) adjustment [58]. Terms with FDR less than or equal to 0.1 were considered as significantly enriched. ErmineJ requires a microarray annotations file that relates array identifiers (Genbank and TUG ids) to Gene Ontology codes and a GO term definition file (gene_ontology.obo), available from the Gene Ontology Web site. Note that ErmineJ observes the "True Path" rule of the Gene Ontology in that annotation with a child GO term implies annotation by all its ancestor terms [33]. Thus, parental terms that are not explicitly cited in the microarray annotations file may be found to be significantly-enriched. To create the microarray annotations file, we performed a provisional GO annotation of the *C. richardii *cDNAs (Genbank ids) using results from a prior blastx analysis in which the *C. richardii *sequences were searched against an *Arabidopsis thaliana *protein sequence database. GO terms associated with the putative *A. thaliana *homologs identified by the blastx analysis were transferred to the *C. richardii *clones. GO annotations for *A. thaliana *were obtained from the Gene Ontology Web site in November, 2006. The microarray annotation file used with ErmineJ is available as Supplmentary Data File 2. For the GO over-representation analysis, cluster members were compared with the full set of TUGs represented on the array.

## Authors' contributions

JY and CC conceived of the study. JY implemented this algorithm and carried out data analysis under the guidance of CC and supervision of SJR. CC, JY, and YSH developed the mathematics underlying the shrinkage correlation coefficient. JY wrote the majority of the manuscript. MLS provided the *C. richardii *replicated microarray data and biological interpretations of the clustering results of the *C. richardii *microarray data. AL performed the Gene Ontology analysis. All authors contributed to, read and approved the final manuscript.

## Supplementary Material

Additional file 1 Data set showing time course of transcript abundance changes during germination of *C*. *richardii *spores for identification of ten samples that might be associated with systematic biases by one-dimensional hierarchical array clustering and SVD. Click here for file

Additional file 2 Supporting data analysis for *C. richardii *microarray quality control. Click here for file

Additional file 3 Data set showing time course of transcript abundance changes during germination of *C. richardii *spores for identification of the gene-wise bias by SVD. Click here for file

Additional file 4 Data set showing time course of transcript abundance changes during germination of *C. richardii *spores for SCC-based clustering analysis. Click here for file
