# Supplementary material for: Genome-scale cluster analysis of replicated microarrays using shrinkage correlation coefficient
Source: BMC Bioinformatics. 2008 Jun 18;9:288. doi: 10.1186/1471-2105-9-288 (PMC2459189; doi:10.1186/1471-2105-9-288)
Supplement: Additional file 2 — Supporting data analysis for C. richardii microarray quality control. [file 1471-2105-9-288-S2.doc]

**Singular Value Decomposition (SVD)**

We used SVD to uncover the artifacts in the data set that were caused by comparison

of different biological replicates and prints of arrays. Let *X* denote an $m\times n$ real matrix with $m\geq n$, the singular value decomposition of the rectangular matrix *X* is a

factorization of the form,

$X=USV^{T}$, [1]

where *U* is an $m\times n$ orthogonal matrix, *S* is an $n\times n$ diagonal matrix with non-negative entries in non-increasing fashion, and $V^{T}$is the transpose of *V*, an $n\times n$ orthogonal matrix. The diagonal entries of *S* are the singular values. The columns of *U* and the rows of $V^{T}$are called the left- and right-singular vectors, respectively. The singular vectors form orthonormal bases. Each left-singular vector is mapped onto the corresponding right-singular vector with the corresponding singular value. Also, each left-singular vector (or right-singular vector) is completely uncorrelated with all the

other left-singular vectors (or right-singular vectors). The fraction of singular value,

$p_{i}=S_{i}^{2}/\sum_{j=1}^{n} S_{j}^{2}$, [2]

indicates the relative variance captured by the *i*th singular value (and the

corresponding left- and right-singular vectors). Following the convention of [1], we refer to the left-singular vectors as eigenarrays, the right-singular vectors as

eigengenes, and the singular values as eigenexpressions. All of the SVD analyses

were implemented with MATLAB (MathWorks, Inc., Natick, MA).

**Quality Control – Array Elimination by Hierarchical Clustering and SVD**

We first applied unsupervised agglomerative hierarchical array clustering to measure the relative similarity among the replicates across all the arrays for each

developmental time point comparison. The hierarchical clustering analyses were carried out simultaneously but separately on the four different time point comparison groups represented by the entire 34 arrays. Figure 1a shows four distinct dendrograms derived from unsupervised hierarchical clustering analysis using the traditional similarity metric, uncentered correlation [2] and average linkage [3]. The biological replicates in each of the four pairwise time point comparison groups were sorted according to the degree of similarity in expression patterns across all TUGs by one-dimensional array clustering.


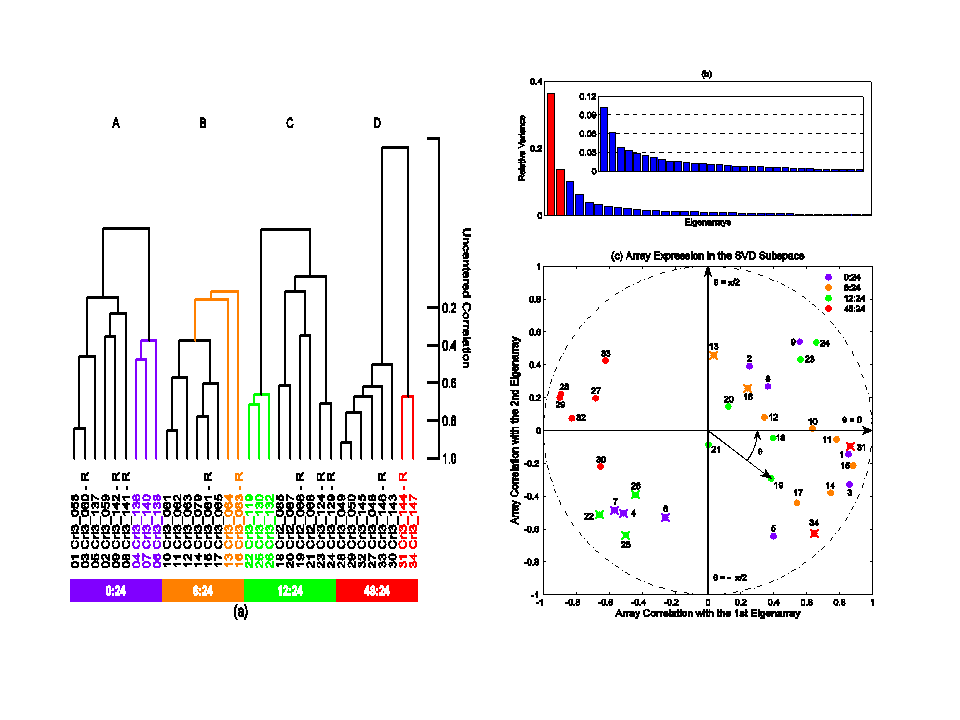


**Figure 1.** (a) Unsupervised hierarchical clustering of the 572 filtered TUGs across 34 arrays. The dendrograms describe the degree of relatedness between arrays, with shorter branches denoting a higher degree of similarity. Under the dendrograms, the horizontal colored boxes delimit four pairwise time point comparison groups. A) 0:24 hr (*violet box*). B) 6:24 hr (*orange box*). C) 12:24 hr (*green box*). D) 48:24 hr (*red box*). Sample names and branches of the outlining samples representing experimental artifacts for each of the five dendrograms are similarly color coded. For each individual group, the samples with name “- R” denote dye-swap replicates. The scale to the right of the dendrograms depicts the uncentered correlation coefficient represented by the length of the dendrograms branches connecting pairs of nodes. (b) Relative variance captured by each eigenarray in the SVD-reduced “*Eigenarrays”* space. The relative variance captured by the first two significant eigenarrays are depicted by red bars, and the remainders by blue bars. (c) Array expression in the two-dimensional SVD subspace. Array correlation with the first eigenarray (*x*-axis) vs. that with the second eigenarray (*y*-axis). The dashed circle outlines normalized array expression in the two-dimensional SVD subspace. The total 34 colored dots denote all of the arrays from the four pairwise time point comparison groups: 0:24 hr (*violet*), 6:24 hr (*orange*), 12:24 hr (*green*), 48:24 hr (*red*). The ten crossed dots denote the samples that might be correlated with systematic biases within the original 34 arrays.

In dendrogram A, all the three reverse replicates (dye-swapped samples) were clustered together with the 1^st^, 2^nd^ and 5^th^ replicates, while the remaining three replicates formed a distinct small cluster. Since the dye-swapped samples were used both as a source of replication and as a control of dye specific bias, grouping the three reverse replicates together with the other three replicates indicates these six samples are most similar to each other and are sufficient for further analysis of the 0:24 hr time point comparison group. The 4^th^, 6^th^ and 7^th^ replicates could be excluded from the further analysis. Dendrogram B shows that while six of the eight 6:24 hr replicates formed a group, including one dye-swapped sample, two arrays were not included in this group. These two arrays, represented by red branches, have very low mean correlation coefficients (~0.11 and ~0.14, respectively) with others. These low correlations suggest that these corresponding samples should not be included in the further analysis. In dendrogram C, 12:24 hr array replicates were split into two distinct subgroups, one including all the three dye-swapped samples and the 18^th^, 20^th^, and 21^st^ replicates, the other composed of only three regular replicates. Grouping the three dye-swapped samples together with three regular replicates indicates these six samples share sufficient similarities for further analysis of the 12:24 hr time point comparison. 48:24 hr replicates were compared in dendrogram D. One dye-swapped sample and one other replicate clustered on a branch distinct from all other samples. The mean correlation coefficients between each of these two samples and the rest are -0.52 and -0.41, respectively, which strongly suggest there are systematic biases existing during the corresponding hybridizations. As a whole, we kept replicates that have higher degrees of similarity from each of the four groups. The uncovered ten samples that have less similarities with other samples might be associated with systematic biases and should be removed from the original data set for further analysis.

To provide further support for the finding of ten samples that might be associated with systematic biases, and to explore the relationships among replicates in each time point comparison group, we performed SVD on the original 34 arrays. SVD is an important factorization of a rectangular matrix and can linearly reduce the input data set of high dimensionality to a lower-dimensional space, which still captures a large fraction of the variance present in the original data [4]. It has seen wide use in the analysis of gene expression data and has proved useful in linear modeling of gene expression [1, 5], cell sample and gene classification [6], gene network modeling [7], experimental artifact uncovering [6, 8]. By projecting the expression arrays to the most significant eigenarrays, SVD can classify the arrays into different groups based on their correlations with these eigenarrays. Arrays with similar expression patterns but with different amplitudes can appear to cluster more tightly. SVD has proven to be a useful method for classifying expression arrays into different cell cycle stages of *S. cerevisiae* [1, 5]. Here, we reduced the original “$TUGs\times Arrays$” space (denoted by *X* in Eq. 1; Supporting Information) to the “*Eigenarrays”* space (denoted by *U* in Eq. 1; Supporting Information) which spans the space of the array expression profiles. In the SVD-reduced “*Eigenarrays”* space, we calculated the relative variance $p_{i}$ (Eq. 2; Supporting Information) captured by each eigenarray. As shown in Figure 1b, the first and second eigenarrays accounted for 50% of the total variance ($p_{1}=36\%$ and $p_{2}=14\%$, respectively) of the data, and might be used to approximate the gene expression changes observed throughout the early stages of gametophyte development of *C. richardii*.

We further investigated the array expression profiles projected to the normalized two-dimensional SVD subspace that is spanned by the first two eigenarrays (Figure 1c). The ten samples identified above that might be associated with systematic biases (Figure 1a) are noticeably separated from the rest of the samples in each time point comparison group. For example, for the 48:24 hr time point comparison group, the arrays separated into two distinct groups: one included 6 arrays which were all anticorrelated with the first and most significant eigenarray, and the other 2 arrays (31^st^ and 34^th^ arrays in Figure 1a) represented by two crossed red dots were both correlated with the first eigenarray and anticorrelated with the second eigenarray. We infer that these 31^st^ and 34^th^ arrays represent the experimental artifacts caused by the hybridizations. This finding corroborates the hierarchical array clustering result shown in dendrogram D in Figure 1a. In the 0:24 hr and 12:24 hr time point comparison groups, all the dubious arrays outlined previously in Figure 1a are clustered together in the third quadrant, while the remaining arrays from those two time point comparison groups spread in the first and fourth quadrants. For the 6:24 hr time point comparison group, although all of the arrays are in the first and fourth quadrants, the 13^th^ and 16^th^ arrays outlined previously in Figure 1a tend to be away from the others. This independent assessment made by SVD is consistent with the above finding by one-dimensional hierarchical array clustering, further indicating those ten samples might be correlated with systematic biases in the hybridizations and most likely represent experimental artifacts.

Furthermore, the array expression profiles projected to the normalized two-dimensional SVD subspace show that arrays from the first three time point comparison groups (0:24 hr, 6:24 hr, and 12:24 hr) express in the first and fourth quadrants in the entire two-dimensional SVD subspace, while the arrays from the 48:24 hr group express in the second and third quadrants. Their opposite correlations with the most significant eigenarray suggest that the first three time point comparison groups may have similar expression profiles which are opposite with that of the 48:24 hr time point comparison group.

**References**

1. Alter O, Brown PO, Botstein D: **Singular value decomposition for genome-wide expression data processing and modeling**. *PNAS* 2000, **97**(18):10101-10106.

2. D'Haeseleer P: **How does gene expression clustering work?** *Nature Biotechnology* 2005, **23**(12):1499-1501.

3. Eisen MB, Spellman PT, Brown PO, Botstein D: **Cluster analysis and display of genome-wide expression patterns**. *PNAS* 1998, **95**(25):14863-14868.

4. Golub GH, Van Loan CF: **Matrix Computations**, 3rd edition edn. Baltimore: The Johns Hopkins University Press; 1996.

5. Holter NS, Mitra M, Maritan A, Cieplak M, Banavar JR, Fedoroff NV: **Fundamental patterns underlying gene expression profiles: Simplicity from complexity**. *PNAS* 2000, **97**(15):8409-8414.

6. Nielsen TO, West RB, Linn SC, Alter O, Knowling MA, O'Connell JX, Zhu S, Fero M, Sherlock G, Pollack JR *et al*: **Molecular characterisation of soft tissue tumours: a gene expression study**. *Lancet* 2002, **359**(9314):1301-1307.

7. Yeung MKS, Tegner J, Collins JJ: **Reverse engineering gene networks using singular value decomposition and robust regression**. *PNAS* 2002, **99**(9):6163-6168.

8. Klevecz RR, Bolen J, Forrest G, Murray DB: **From the Cover: A genomewide oscillation in transcription gates DNA replication and cell cycle**. *PNAS* 2004, **101**(5):1200-1205.
